# Angiogenin contributes to bladder cancer tumorigenesis by DNMT3b-mediated MMP2 activation

**DOI:** 10.18632/oncotarget.10097

**Published:** 2016-06-15

**Authors:** Rafael Peres, Hideki Furuya, Ian Pagano, Yoshiko Shimizu, Kanani Hokutan, Charles J. Rosser

**Affiliations:** ^1^ University of Hawaii Cancer Center, Clinical & Translational Research Program, Honolulu, HI, USA; ^2^ University of Hawaii Cancer Center, Cancer Prevention and Control Program, Honolulu, HI, USA; ^3^ Department of Molecular Biosciences and Bioengineering, University of Hawaii at Manoa, Honolulu, HI, USA

**Keywords:** angiogenin, bladder cancer, DNMT3b, MMP2

## Abstract

Epigenetic-mediated gene activation/silencing plays a crucial role in human tumorigenesis. Eliciting the underlying mechanism behind certain epigenetic changes is essential for understanding tumor biology. Previous studies in human cancers revealed an unrecognized interplay between Angiogenin (ANG) and matrix metalloproteinase-2 (MMP2) leading to pronounced tumorigenesis. Here we provide multiple lines of evidence further indicating ANG oncogenic potential. ANG expression resulted in the hypomethylated state of the MMP2 gene, which led to increased gene expression of MMP2. More than that, our global DNA methylation microarray analysis showed that gene manipulation of ANG affected a variety of pathways, such as cell migration, angiogenesis and specifically, tumor suppressor genes. Mechanistically, ANG negatively regulated DNA methyltransferase 3b (DNMT3b) enzymatic activity by down-regulating its expression and inhibiting its recruitment to the MMP2 promoter. Consistent with this, ANG-MMP2 overexpression and DNMT3b underexpression correlated with reduction in disease free survival of human bladder cancer patients. Together, the results continue to establish ANG as an oncoprotein and further reveal that ANG contributes to oncogenesis by the activation of MMP2 through modulation of DNMT3b functions.

## INTRODUCTION

Changes in cancer cell transcriptome can be driven by genetic and epigenetic alterations. An important epigenetic process that is implicated in mammalian development is the methylation of cytosine within the context of a simple dinucleotide site, CpG. DNA methylation is an epigenetic process that can alter gene expression without altering DNA sequence[1]. Specifically, DNA methylation plays a key role in controlling several biological processes including: X chromosome inactivation, genomic imprinting, genomic stability and chromatin structure [2]. DNA methylation controls these biologic processes by its ability to repress the gene transcription. In addition to these normal biologic processes, deregulated DNA methylation can also be a major driver of pathologic conditions such as neurological and autoimmune diseases, as well as cancers [3].

During tumorigenesis, global DNA methylation patterns are altered. While DNA CpG hypermethylation has been shown to result in abnormal silencing of several tumor suppressor genes, DNA hypomethylation result in abnormal activation of several oncogenes [3]. Three cytosine DNA methyltransferase enzymes (DNMTs) catalyze DNA methylation at the 5′ cytosine of CpG sites. DNMT1 is the most abundant, and functions as a maintenance methyltransferase to establish normal methylation patterns during embryogenesis, and to reproduce these methylation patterns during cellular replication as well as *de novo* methylation following DNA damage [4]. Previous report has demonstrated that DNMT1 expression is increased in bladder cancer [5]. DNMT3a and DNMT3b function as *de novo* methyltransferases to establish new methylation patterns by targeting normally unmethylated CpG sites on gene promoters [6]. However, limited reports are available describing DNMT3a and DNMT3b expression in bladder cancer [7].

Previously, we reported a novel interplay between angiogenin (ANG), a potent mediator of angiogenesis, and matrix metalloproteinase-2 (MMP2) in human bladder tumors [8]. Specifically, we showed that forced ANG overexpression in benign human bladder UROtsa cell line induced cellular survival, proliferation, endothelial tube formation and xenograft angiogenesis and growth by mediating the MAPK/ERK-MMP2 axis [8]. ANG, a 14.2 kDa polypeptide member of the RNase A superfamily, which was originally isolated from conditioned media of HT-29 colon adenocarcinoma cells, is an understudied angiogenic factor in human cancers [9]. Its expression is noted to be up-regulated in some cancers [10–14]. Elevated ANG expression has been linked to higher rates of invasive and higher-grade bladder cancers as well as worse clinical outcomes [15].

ANG is taken up by endothelial cells by receptor-mediated endocytosis and rapidly translocates to the nucleus, a process essential for its angiogenic activity. In the nucleus, ANG cleaves tRNA and promotes ribosomal RNA transcription affecting a wide variety of responses including proliferation, cell migration and invasion, and tube formation [16]. Although we previously reported on the interplay between ANG-ERK1/2-MMP2 and identified ANG as an oncoprotein, there is no direct evidence as to how ANG regulates MMP2 expression and exert its effects on cellular survival and proliferation. We hypothesized that ANG itself or a transcription factor regulated by ANG may play a direct role in MMP2 gene transcription.

MMP2 is a member of the MMP enzyme family that is capable of cleaving components of the extracellular matrix and is involved in signal transduction. It is thought to be involved in multiple biologic processes including neurogenesis, endometrial menstrual breakdown, regulation of vascularization, and metastasis [17]. Here, we describe how ANG regulates MMP2 expression by regulating methylation status by its effects on DNMT3b, and thus the transcriptional capability, of MMP2. Furthermore, when ANG, MMP2 are overexpressed and DNMT3b expression reduced in bladder tumors, there is a significant reduction in disease specific survival, thus attesting to ANG, DMT3b and MMP2 ability to further risk stratify patients that may require a more aggressive, even personalized, management plan. Our findings pave the way to advance our understanding of human bladder tumor biology and confirm ANG as a potential biomarker and as a target for therapeutic intervention.

## RESULTS

### ANG regulates the transcription of MMP2 gene

**Figure 1:**
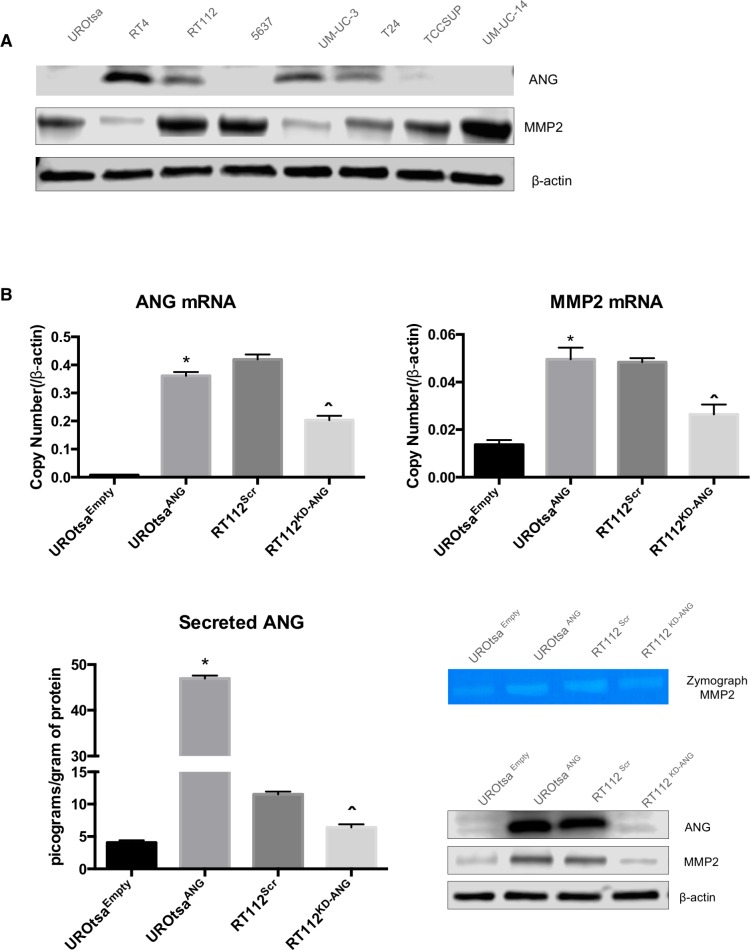
ANG overexpression in human bladder cell lines leads to MMP2 expression **A.** ANG and MMP2 levels were evaluated in UROtsa, RT4, RT112, 5637, UM-UC-3, T24, TCCSUP, and UM-UC-14 by immunoblotting. **B.** UROtsa cells were stably transfected with empty vector (UROtsa^Empty^) or ANG expression plasmid (UROtsa^ANG^), while RT112 cells were transiently transfected with negative control siRNA (RT112^Scr^) or siRNA directed at ANG mRNA (RT112^KD-ANG^). The mRNA levels were analyzed by quantitative RT-PCR and protein levels were analyzed by immunoblotting analysis. β-Actin was used as an internal loading control. **C**. ANG ELISA assay also confirmed altered secretion of ANG in the same cell lines. Zymogen assays were performed to assess the activity of MMP2. Three independent experiments were performed in triplicate. *p < 0.05; *vs.* UROtsa^Empty^, and ˆ*p* < 0.05; *vs.* RT112^Scr^.

Evaluation of a panel of human bladder cell lines (UROtsa, RT4, RT112, 5637, UM-UC-3, T24, TCCSUP and UM-UC-14) demonstrated a range of ANG and MMP2 expression levels (Figure 1A). To test the influence of ANG, we created stably transfected ANG-overexpressing UROtsa cells (UROtsa^ANG^ and control UROtsa^Empty^), and transiently transfected RT112 cells with specific siRNAs to knockdown ANG (RT112^KD-ANG^ and control RT112^Scr^). Cellular protein expression of ANG and MMP2, mRNA expression of ANG and MMP2, secreted ANG protein measured by ELISA and active MMP2 (measured by Zymograph) were shown in Figure 1B. To confirm the data from in RT112KD-ANG from siRNA, we performed independent experiments using 3 additional siRNA against ANG (Supplemental Figure 1). Comparable gene silencing efficacy was achieved, inducing similar changes in gene expression profiles of MMP2. Thus as we previously described, high ANG expression correlated with an increase in active MMP2.

### ANG expression leads to a hypomethylation of MMP2 gene as well as global methylation changes

**Figure 2:**
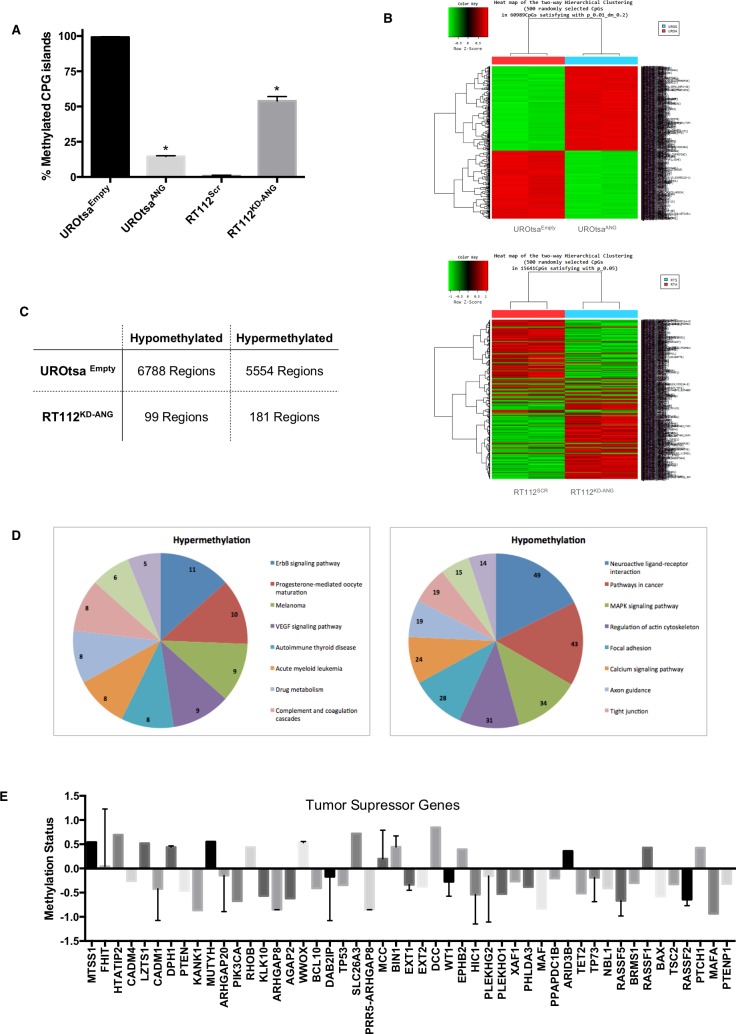
Epigenetic changes in the MMP2 gene promoter and globally in human bladder cells expressing ANG **A.** Graphical analysis of CpG methylation pattern in the MMP2 promoter gene. The percentages of methylated CpGs in UROtsa^ANG^, UROtsa^Empty^, RT112^KD-ANG^ and RT112^Scr^ cells were noted. *p < 0.01. **B.** Hierarchical cluster analysis of methylation data profiling in UROtsa^ANG^, UROtsa^Empty^ and RT112^KD-ANG^ and RT112^Scr^ cells. Each row represents a methylated gene and each column a sample. The scale represents standard deviations from the mean after a *Z*-transformation of signal values of a gene across all samples. *Red* represents a higher level of gene expression and *green* a lower level, relative to the mean across all samples for each gene. **C.** Illumina chip analysis of the changed DNA methylation patterns of MMP2. Hypermethylation and hypomethylation: the status of MMP2 DNA methylation in UROtsa^ANG^ compared to UROtsa^Empty^, and RT112^KD-ANG^ compared to RT112^SCR^. **D.** Pathway analysis of the identified genes with changed promoter DNA methylation status. Eleven of the most significantly enriched pathways were plotted for genes of hypermethylation (left panel) or hypomethylation (right panel) in UROtsa^ANG^ compared to UROtsa^Empty^. **E.** Methylation status of 48 tumor suppressor genes within the Illumina whole genome methylation array in UROtsa^ANG^ compared to UROtsa^Empty^.

To explore the mechanism by which ANG regulates MMP2 expression, we investigated whether ANG is functionally linked to DNA methylation-based transcriptional regulation. First, we quantitatively assessed the methylation status of the MMP2 promoter. The result showed that high expression levels of ANG in UROtsa^ANG^ and RT112^Scr^ were associated with a reduction in percentage methylated CpG islands of the MMP2 promoter compared to UROtsa^Empty^ and RT112^KD-ANG^, respectively (Figure 2A). The alteration of genome-wide DNA methylation in these cells was then analyzed using Illumina whole genome methylation array, a high sample throughput solution for epigenome-wide association studies that have been used multiple times in cancer epigenetic research [18, 19]. ANG expression was noted to be associated with a change in global DNA methylation levels compared to control cells (Figure 2B). We found that a total of 12,342 regions in the genome had significant changes in methylation patterns between UROtsa^Empty^ and UROtsa^ANG^, including 6788 hypomethylated regions and 5554 hypermethylated. Similarly when comparing RT112^Scr^ and RT112^KD-ANG^ cells, a total of 280 regions had significant changes in methylation patterns including 99 hypomethylated regions and 181 hypermethylated (Figure 2C). The genes affected by those regions were then classified into various cellular signaling pathways using Molecule Annotation System software. Ten pathways enriched with most genes with hypermethylation or hypomethylation in UROtsa^ANG^ cells were compared to UROtsa^Empty^ cells (Figure 2D). We also analyzed the methylation status of 48 tumor suppressor genes within the Illumina whole genome methylation array. Sixteen hypermethylated genes and 32 hypomethylated genes were identified (Figure 2E).

Next, angiogenesis PCR array data and Illumina whole genome methylation array data were integrated in order to correlate the methylation status to gene expression status of 18 targets that were consistently associated with ANG in UROtsa and RT112 cell lines (Supplemental Table 1), confirming the ability of ANG to regulate gene expression through changes in methylation status. For example in UROtsa^ANG^ cells (compared to UROtsa^Empty^), MMP2 gene was hypomethylated and overexpressed, while in RT112^KD-ANG^ (compared to RT112^Scr^), MMP2 gene had a mixed hypomethylated and hypermethylated pattern leading to under expression of MMP2. Taken together, these results support the idea that ANG possesses the ability to change the methylation status of MMP2 gene as well as numerous other genes, in this way affecting their expression level.

### Silencing DNMT3b in the presence of ANG leads to increased MMP2 expression

**Figure 3:**
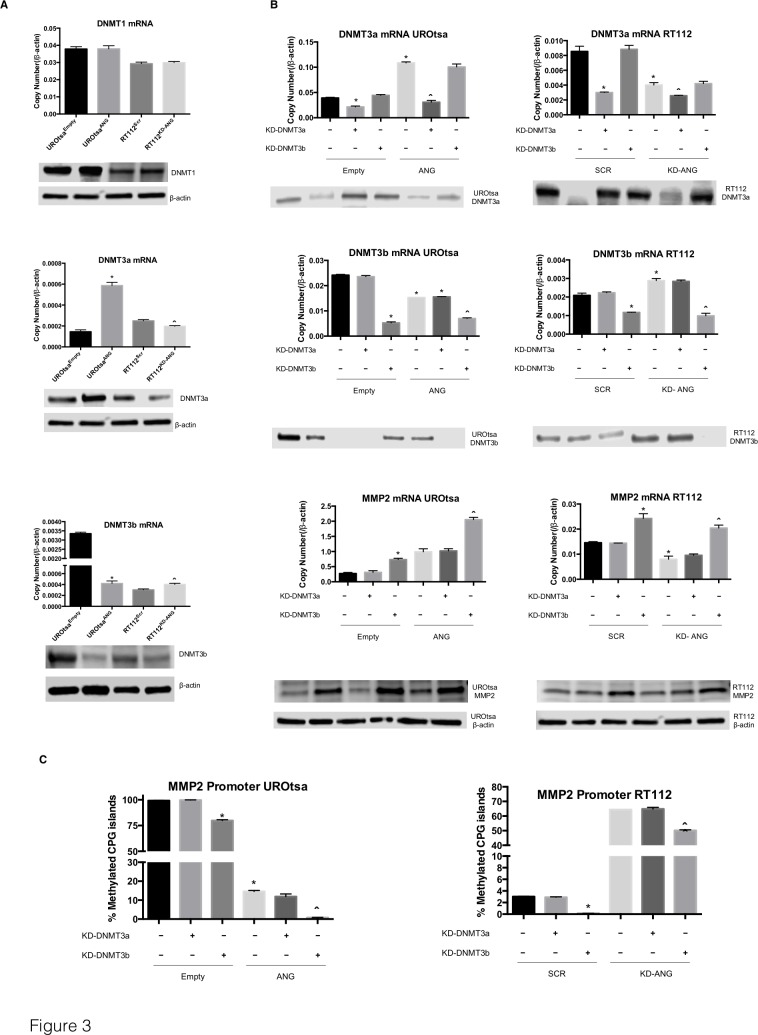
DNMT3b and MMP2 are highly regulated by ANG overexpression **A.** mRNA and protein expression levels of DNMT1, DNMT3a and DNMT3b were assessed in UROtsa^ANG^, UROtsa^Empty^, RT112^KD-ANG^ and RT112^Scr^ cells. *p < 0.05; *vs.* UROtsa^Empty^, and ˆ*p* < 0.05; *vs.* RT112^Scr^. β-actin was used as an internal control. **B.** The effects of DNMT3a and DNMT3b knockdown on MMP2 mRNA and protein levels were assessed. The mRNA levels were analyzed by qRT-PCR and protein levels were analyzed by immunoblotting analysis. β-actin was used as an internal control. **C.** The effects of DNMT3a and DNMT3b knockdown on percentages of methylated CpG islands of the MMP2 promoter were assessed using Epitect methyl II PCR array. Depicted is one of three independent experiments, which were performed in triplicate. *p < 0.05; *vs.* Empty-Scr or Scr-Scr, and ˆ*p* < 0.05; *vs.* ANG-Scr or KD-ANG - Scr.

To better understand the regulation of the MMP2 gene methylation status by DNMTs, we analyzed the mRNA and protein expression of DNMT1, DNMT3a and DNMT3b in UROtsa^Empty^, UROtsa^ANG^, RT112^Scr^ and RT112^KD-ANG^ bladder cell lines (Figure 3A). No difference in the expression of DNMT1 was noted among the cell lines. Therefore, analysis of DNMT1 was not carried out in subsequent experiments. However, expression of DNMT3a was increased 3-folds in UROtsa^ANG^ cells compared to control cells, while its expression was reduced 20% in RT112^KD-ANG^ cells compared to control cells. On the other hand, overexpression of ANG in UROtsa cells reduced DNMT3b expression by 87.5% compared to control cells, while increasing its expression by 25% in RT112^KD-ANG^ compared to control cells. For all of the cell lines, protein expression of the DNMTs correlated to gene expression.

To determine if DNMT3a and/or DNMT3b mediate ANG ability to regulate the MMP2 gene methylation status, we individually silenced DNMT3a and DNMT3b by siRNA and monitored the mRNA and protein expression of MMP2 in UROtsa^Empty^, UROtsa^ANG^, RT112^Scr^ and RT112^KD-ANG^ (Figure 3B). Knockdown of the DNMT3a did not alter MMP2 expression in any of the cell lines. Interestingly, knockdown of DNMT3b in UROtsa^Empty^ caused an enhancement of MMP2 expression comparable to the impact of ANG forced overexpression. MMP2 expression was increased by the combination of ANG overexpression and DNMT3b knockdown, with a 7.5 fold increase. Similarly, DNMT3b knockdown in RT112 cells induced MMP2 expression. Notably, ANG knockdown reduced MMP2 expression, while DNMT3b knockdown rescued MMP2 expression inhibited by ANG knockdown. To further confirm these results, we analyzed the percentage of methylated CpG islands of the MMP2 promoter when inhibiting the methyltransferases by siRNA (Figure 3C). Supporting the MMP2 expression results, DNMT3b knockdown induced a hypomethylation of the MMP2 promoter, while DNMT3a knockdown did not change the methylation status. Collectively, these results strongly suggest that ANG positively regulates MMP2 expression by changing the methylation status of MMP2 promoter *via* DNMT3b.

### Expression of ANG and MMP2 in human bladder tumors adversely effects disease free survival

**Figure 4:**
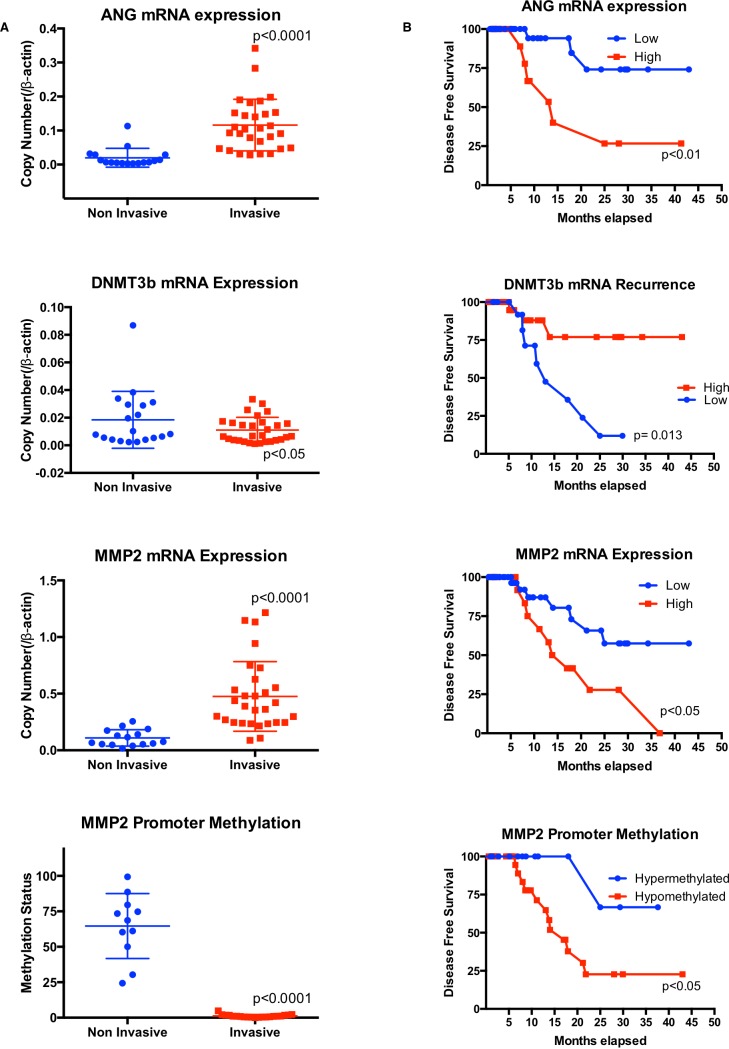
High ANG levels are associated with low DNMT3b and high MMP2 levels, which are related to poor prognosis in human bladder tumors **A.** Analysis of ANG, DNMT3b and MMP2 mRNA levels and methylation status (percentages of methylated CpG islands) of MMP2 gene in muscle invasive bladder cancer and non-muscle invasive bladder cancer. **B.** Kaplan-Meier survival rate analysis for ANG, DNMT3b and MMP2 expression levels and methylation status of MMP2 gene in human bladder cancer. Tumor samples with patient history (see Table 1) were used for survival analysis of A-D.

To further define the role of ANG, DNMT3b and MMP2 in tumorigenesis, we collected 78 fresh bladder cancer samples from bladder cancer patients and extracted DNA and RNA for analysis. Demographic and clinicopathologic features of the study cohort are reported in Table 1. We first analyzed ANG, DNMT3b and MMP2 mRNA levels in human bladder cancer tissues by quantitative RT-PCR. Muscle invasive bladder cancer specimens had increased levels of ANG, MMP2 and decreased levels of DNMT3b compared to non-muscle invasive bladder cancer. Moreover, the muscle invasive bladder specimens were noted to have a reduction in the methylation status of the MMP2 promoter compared to the non-muscle invasive bladder cancer (Figure 4A). One large public dataset (GDS4456) [20] was obtained from the GEO website and analyzed for ANG and MMP2 expression (Supplemental Figure 2). Mean relative mRNA levels for both ANG and MMP2 in muscle invasive bladder cancer were significantly elevated compared to the mean relative mRNA levels for ANG and MMP2 in non-muscle invasive bladder cancer, thus confirming our results.

In addition, statistical analyses noted Spearman correlation coefficient of 0.802 when the relative levels of ANG was plotted against MMP2 and −0.689 when the relative levels of ANG was plotted against methylation of MMP2 promoter. More importantly, disease free survival was adversely and significantly effected if: a) ANG was overexpressed (median survival 2.0 years in high ANG expressing tumors *vs.* 3.0 years in low ANG expressing tumors, *p* = 0.009), b) DNMT3b was underexpressed (median survival 2.4 in high DNMT3b expressing tumors *vs.* 2.1 years in low DNMT3b expressing tumors, *p* = 0.013), or c) MMP2 was overexpressed (median survival 1.9 in high MMP2 expressing tumors *vs.* 2.5 years in low MMP2 expressing tumors, *p* = 0.047). Furthermore, the methylation status of the MMP2 promoter correlated with disease free survival, *i.e.,* hypermethylation of the MMP2 gene had a median survival of 2.05 years, while hypomethylation of the MMP2 promoter had a reduced median survival of 1.44 years, *p* = 0.034 (Figure 4B).

Univariate analysis of key demographic and clinicopathologic features revealed that adverse expression levels of DNMT3b, and overexpression of ANG and MMP2 together with hypomethylation of MMP2 gene promoter represented unfavorable prognostic factors associated with disease free survival (Table 1). On multivariate analysis, adverse expression levels of MMP2-DNMT3b represented unfavorable prognostic factors associated with a reduction in disease free survival (Table 1). Specifically, the concomitant adverse expression of ANG-DNMT3b-MMP2 with hypomethylation of the MMP2 promoter was associated with a hazard ratio [HR] of 17, 95% confidence interval [CI] = 2.624 - 129.04, *p* = 0.006. Thus, the survival analysis further suggests the ANG-DNMT3b-MMP2 axis play a role in bladder cancer progression and thus could hold promise as prognostic markers.

**Table 1:** Analysis of key demographic and clinicopathologic features of patients

				Univariate Analyses					Multivariate Analyses		
	*n*	Median	HR	95% LCL	95% UCL	*p*	Median	HR	95% LCL	95% UCL	*p*
Age (yrs)											
_ 65	20	2.89	1.00				2.49	1.00			
> 65	38	1.71	2.74	0.77	9.82	.12	1.27	3.38	0.86	13.23	.08
Sex											
Female	13	1.48	1.00				1.68	1.00			
Male	45	2.23	0.55	0.17	1.74	.31	1.89	0.77	0.23	2.61	.68
Grade											
Low	4	2.05	1.00				1.26	1.00			
High	53	2.07	0.75	0.15	3.66	.72	2.52	0.27	0.04	2.02	.20
Stage											
NMIBC	19	2.48	1.00				2.26	1.00			
MIBC	38	1.85	1.43	0.49	4.15	.51	1.40	2.21	0.58	8.42	.25
ANG											
Favorable (Low) ( < 0.38 )	27	3.02	1.00				2.46	1.00			
Adverse (High) ( > 0.38 )	21	2.02	1.79	0.50	6.41	.37	2.16	1.68	0.39	7.31	.49
MMP2											
Favorable (Low) ( < 0.25 )	27	2.47	1.00				3.24	1.00			
Adverse (High) ( > 0.25 )	22	1.88	1.49	0.48	4.65	.49	1.58	1.99	0.40	9.94	.40
DNMT3b											
Favorable (High) ( > 0.008 )	21	2.43	1.00				2.04	1.00			
Adverse (Low) ( < 0.008 )	26	2.10	1.34	0.35	5.11	.67	2.44	0.97	0.24	4.01	.97
Methylation											
Favorable (High) ( > 1.12 )	24	2.05	1.00				1.42	1.00			
Adverse (Low) ( < 1.12 )	20	1.44	1.73	0.54	5.58	.36	1.39	1.13	0.23	5.65	.88
ANG + MMP2											
Favorable (one or both)	30	2.64	1.00				2.95	1.00			
Both Adverse	15	1.63	2.06	0.57	7.47	.27	1.84	1.93	0.34	10.95	.46
ANG + DNMT3b											
Favorable (one or both)	36	2.92	1.00				2.88	1.00			
Both Adverse	9	1.61	2.58	0.73	9.05	.14	1.88	2.41	0.56	10.27	.24
ANG + Methylation											
Favorable (one or both)	32	2.22	1.00								
Both Adverse	6	1.41	2.18	0.45	10.67	.34					
MMP2 + DNMT3b											
Favorable (one or both)	37	2.80	1.00				3.90	1.00			
Both Adverse	10	1.10	5.03	1.50	16.90	.01	1.36	5.77	1.21	27.61	.03
MMP2 + Methylation											
Favorable (one or both)	30	2.11	1.00								
Both Adverse	8	0.67	10.07	2.05	49.39	.004					
DNMT3b + Methylation											
Favorable (one or both)	26	2.31	1.00								
Both Adverse	11	1.11	3.01	0.81	11.13	.10					
ANG + MMP2 + DNMT3b											
Favorable (one or more)	39	2.87	1.00				3.89	1.00			
All Adverse	6	1.11	4.56	1.27	16.43	.02	1.35	5.25	0.89	30.87	.07
ANG + MMP2 + Methylation											
Favorable (one or more)	31	2.11	1.00								
All Adverse	4	0.56	17.00	2.24	129.04	.006					
ANG + DNMT3b + Methylation											
Favorable (one or more)	32	2.11	1.00								
All Adverse	3	0.52	17.00	2.24	129.04	.006					
MMP2 + DNMT3b + Methylation											
Favorable (one or more)	31	2.11	1.00								
All Adverse	6	0.65	10.07	2.05	49.39	.004					
ANG + MMP2 + DNMT3b + Methyl											
Favorable (one or more)	32	2.11	1.00								
All Adverse	3	0.52	17.00	2.24	129.04	.006					

Demographic and clinicopathologic features in a cohort of 78 patients. ANG, DNMT3b and MMP2 mRNA levels and MMP2 gene promoter methylation were investigated. Univariate revealed that adverse expression levels of DNMT3b, and overexpression of ANG and MMP2 together with hypomethylation of MMP2 gene promoter represented unfavorable prognostic factors associated with disease free survival. On multivariate analysis, adverse expression levels of MMP2-DNMT3b represented unfavorable prognostic factors associated with a reduction in disease free survival. The multivariate analyses adjust for the demographic and clinicopathologic variables. The hazard ratio (HR) is estimated from a semiparametric (Cox proportional hazards) model. The median years to recurrence is estimated from a parametric model. Expression variables dichotomized at the full sample medians. Empty cells could not be estimated.

## DISCUSSION

**Figure 5:**
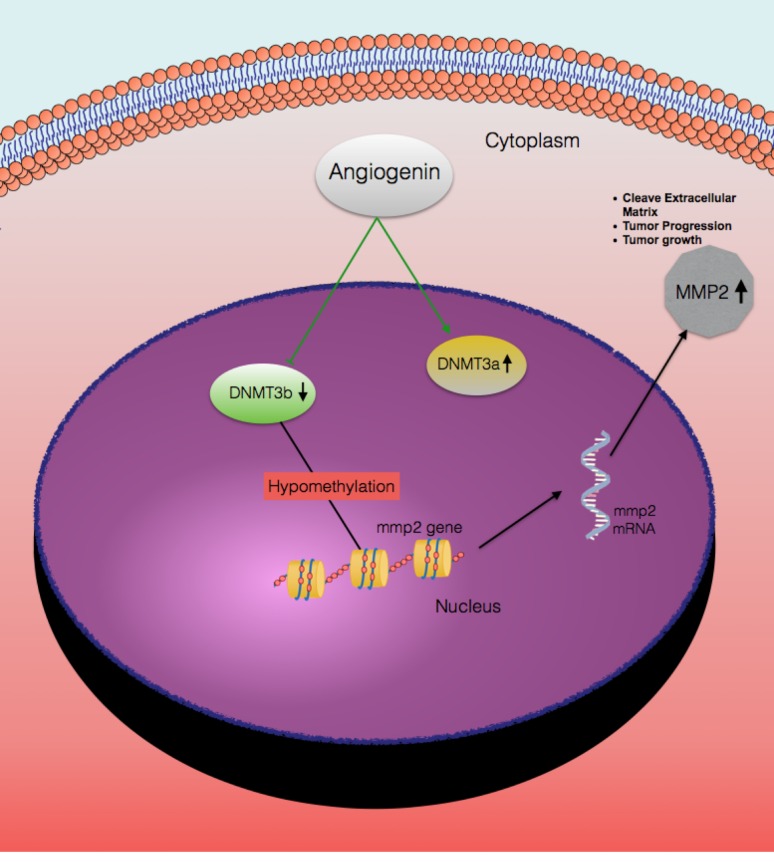
A hypothetical representation of the regulatory pathway underlying ANG-induced MMP2 expression Based on this study, ANG has two different effects on the cells a) induces DNMT3a expression, b) represses DNMT3b expression. Reduced DNMT3b levels lead to hypomethylation of the MMP2 promoter, which induces MMP2 gene transcription. Thus, expression of MMP2 contributes to increase invasive potential of bladder cancer and reduced disease free survival.

Based on our current and published work [8], we have provided *in vitro* and *in vivo* evidence demonstrating the oncogenic activity of ANG. We also demonstrate that ANG can positively regulate MMP2 expression. Specifically, ANG expression represses DNMT3b, causing hypomethylation of the MMP2 promoter leading to increase ability to transcribe the MMP2 gene (Figure 5). Interestingly, expression of ANG, DNMT3b and MMP2 is correlated with disease free survival in human bladder cancer.

We found that ANG regulates methylation status of the MMP2 promoter and that ANG changes genome-wide DNA methylation status. Accumulating evidence suggest that expression of MMPs is primarily regulated at the transcriptional level [21, 22], although the factors governing the expression of specific MMPs in different tissues have yet to be fully defined. The intricate transcriptional regulation of MMPs involves mechanism as promoter polymorphisms, epigenetic regulation [22], and post-transcriptional processes [23]. MMP2 gene promoter hypomethylation (and mRNA overexpression) had been previously described in periapical granulomas [24], but this is the first description in bladder cancer. DNA methylation is a covalent modification of the genome, which plays a key role in regulating chromatin structure but also recruit remodeling enzymes to reposition nucleosomes, thereby modulating processes, such as transcription, DNA repair, replication and recombination [25, 26]. Alterations of DNA methylation pattern are one of the most consistent epigenetic changes in human cancers. The results from this study demonstrated the association among ANG, MMP2 gene promoter methylation and mRNA expression, suggesting that ANG regulates MMP2 expression by regulating DNA methylation of MMP2 promoter. Previous studies have shown the correlation between genome-wide DNA methylation and clinical outcomes [3]. Indeed, our global DNA methylation microarray analysis showed that gene manipulation of ANG affected a variety of pathways, including cell migration, pathways in cancer, angiogenesis and tumor suppressor genes. Most of those tumor suppressor genes also had changes in their expression levels, which correlate to the poor outcome observed in patients with high expression of ANG. Although DNA abnormal methylation had been shown to result in anomalous silencing of several tumor suppressor genes in most types of cancer [3, 27], this is the first report to show ANG ability to induce DNA abnormal methylation leading to bladder tumorigenesis. This anomalistic methylation could be a useful therapeutic target in cancer [28], specially considering that several clinical trials have been developed recently to explore the use of DNA methylation inhibitors in cancer treatment [29, 30].

We report that downregulation of DNMT3b in the presence of ANG leads to increased MMP2 expression. To determine the mechanism by which ANG regulates DNA methylation of MMP2 promoter, we profiled DNMTs levels. DNMT3a and DNMT3b function as *de novo* methyltransferases that play important roles in normal development and diseases, with DNMT3b being able to methylate a broader spectrum of target sequences [6]. DNMT1 has been classically described as the major maintenance methyltransferase [31]. However, there are recent reports that DNMT1 works together with DNMT3b in silencing genes in human cancer cells [32]. Herein we show that ANG overexpression does not change DNMT1 levels, but it does down-regulate DNMT3b and up-regulate DNMT3a. These results corroborate previous study that alterations in the expression of methyltransferases are related to tumor development [7]. The fact that ANG overexpression in the UROtsa cells caused a more conspicuous change for the methyltransferases than the knockdown in RT112 probably explains the differences in the amount CpG islands affected between these cell lines. Furthermore, we also noted that downregulation of DNMT3b, but not DNMT3a induced hypomethylation of the MMP2 promoter. These results reveal ANG oncogenic properties, since it was able to change the methylation status of a diverse array of cancer related genes, specifically MMP2.

MMPs are known to affect extracellular matrix (ECM) remodeling, angiogenesis, apoptosis, epithelial-to-mesenchymal transition and cell proliferation and thus induce aggressive invasion and metastasis of different cancer types, including bladder cancer [33]. In fact, a significant body of literature has described MMP9 to be upregulated in bladder cancer. In two large epidemiologic studies, genetic changes in MMP9 gene were associated with the development of invasive bladder cancer and affected overall survival [34]. Futhermore, the secretion of MMP9 protein was increased in invasive bladder cancers [35]. Data related to MMP2 in bladder cancer is scarce. However in the same setting where urinary MMP9 was elevated, Gerards *et al.* and Sier *et al.* noted MMP2 to be increased. Previously, Miyake *et al.* reported the positive correlation of gene and protein expression of MMP2 in ANG expressing cell lines all leading to tumor growth *via* angiogenesis [8]. In this study, elevated ANG expression was found to lead to the overexpression of MMP2 by hypomethylation of its promoter allowing for more efficient transcription through DNMT3b down regulation. The results suggest that the MMP2 gene is amplified in ANG-overexpressing bladder cancer cells, supporting ANG's oncogenic characteristics. In addition to these bladder cancer cell lines, MMP2 gene amplification may occur in bladder tumors with high ANG levels. In our analysis of a publicly available database, it was suggested that ANG and MMP2 genes are amplified in a subset of bladder cancers. The results analyzed using GEO database also show that muscle invasive bladder cancer specimens had increased levels of ANG and MMP2 compared to non-muscle invasive bladder cancer (Supplemental Figure 3). These findings suggest that the MMP2 gene may be a mechanism underlying ANG overexpression in bladder cancer and thus may promote bladder cancer growth and angiogenesis.

ANG requirement for the cellular survival, proliferation and angiogenesis of ANG-overexpressing bladder cancer cells is in accordance with the oncogenic model in which the growth and survival of cancer cells can be suppressed by the inactivation of a single oncogene [36]. Overexpression of certain oncogenes, such as Ras and Myc, is linked to poor clinical outcomes. The current study demonstrated that, similar to Ras and Myc, high expression of ANG correlated with reduced disease specific survival in a human bladder cancer tumor set (n = 78). Our results indicate that the ANG gene is amplified in human bladder cancer, leading to reduced expression of DNMT3b and increased expression of MMP2. In fact, on univariate analysis, adverse expression of ANG-DNMT3b-MMP2 and hypomethylation of MMP2 promoter was associated with a hazard ratio [HR] of 17.00, (95% confidence interval [CI] = 2.24 - 129.04, *p* = 0.006) the strongest predictor of disease free survival in this study. Thus, it is tempting to speculate that the adverse expression of ANG-DNMT3b-MMP2 may be utilized as a prognostic factor for human bladder cancer. Together, our results suggest the oncogenic properties for ANG in human bladder tumorigenesis.

In summary, we demonstrate that ANG is overexpressed in approximately 45% of bladder tumors. This results in overexpression of MMP2, a reduction in DNMT3b and deep changes in the methylation status genome wide. Such molecular alterations are associated with higher rates of muscle invasive bladder cancers as well as a reduction in disease specific survival, thus attesting to ANG and MMP2 ability to further risk stratify patients that may require a more aggressive, even personalized, management plan. Therefore, the study provides novel evidence that ANG plays an important role in the promoter activation of the tumor oncogenic gene MMP2, which likely contributes to ANG oncogenic activity.

## MATERIALS AND METHODS

### Cells lines

A panel of human bladder cancer cell lines (UM-UC-14, RT112, RT4, 5637, UM-UC-3, TCCSUP) was provided by the Pathology Core of the Bladder Cancer SPORE at MD Anderson Cancer Center. These cells were authenticated by DNA fingerprinting using the AmpFISTR Amplification or AmpFISTR Profiler PCR Amplification protocols (Life Technologies, Carlsbad, CA). The human bladder cancer cell line T24 (ATCC, Manassas, VA) and the benign human bladder cell line UROtsa (gift from Dr. Donald Sens at the University of North Dakota School of Medicine, Grand Forks, ND) were authenticated by Genetic Resources Core Facility at Johns Hopkins (Baltimore, MD). Cell lines were maintained in DMEM or RPMI 1640 media as previously described [37]. All cell lines were confirmed to be mycoplasma free.

### Stable transfection of cell lines

UROtsa cells, which had reduced levels of ANG, were stably transfected to express ANG (UROtsa^ANG^ and UROtsa^EMPTY^) as previously described [8]. Stable transfectants were maintained in media containing 1,200 μg/ml of G418 for UROtsa clones.

### Transient transfection of small interfering RNA (siRNA)

RT112 cells, which had elevated levels of ANG, were transfected with synthesized commercial ANG or scrambled (Scr) negative control siRNA (Life Technologies) using 6 well plates with a 100-pmol of siRNA and 9 μl of INTERFERin (Polyplus-transfection Inc., NY, USA) for 72 hrs according to manufacture's instruction. RT112 cells treated with siRNA for ANG or Scr are named as RT112^KD-ANG^ and RT112^Scr^, respectively.

RT112 and UROtsa cells were transfected with synthesized commercial siRNA for MMP2, DNMT3a, DNMT3b or Scr siRNA (Life Technologies) for 48 or 72 hrs. For promoter methylation analysis, cells were incubated for 48 hrs after the first siRNA transfection, then performed second transfection (following the same protocol) and incubated for extra 48 hrs.

### Immunoblotting

Cell lysis and immunoblotting were performed using standard protocols as previously described [8]. Antibody details are available in Supplementary Material. The immunoblots were visualized using Clarity Western enhanced 512 chemiluminescence substrate (Bio-Rad, Hercules, CA) and a digital darkroom (C-Digit Blot Scanner, Li-Cor Biosciences, Lincoln, NE).

### Quantitative reverse transcriptase-PCR

Total RNA extraction was performed using RNeasy mini kit according to manufacturer's instruction (Qiagen, Germantown, MD). cDNA was synthesized using qScript cDNA SuperMix (Quanta Biosciences, Gaithersburg, MD). Real-time PCR was performed with MyiQ2 Two-Color Real-Time PCR Detection System (Bio-Rad). The standard real-time PCR reaction volume was 20 μl, and consisted of 10 μl of PerfeCTa SYBR Green FastMix (Quanta Biosciences), 7 μl RNAse-free H_2_O, 1 μl (final concentration of 1 μM) forward primer, 1μl (1 μM) reverse primer and 1 μl (0.5 ng/μl) cDNA. All reactions were performed in triplicate. Primer sets can be found in Supplementary Material. Relative concentrations of mRNA levels were calculated after normalization to β-actin using absolute quantification.

### Measurement of secreted ANG by ELISA

Cells were seeded onto 6-well plate at a density of 1.5 × 10^5^ cells/well. After 48 hrs, the conditioned media were collected and centrifuged to remove any dead or floating cells. Conditioned media was analyzed by ELISA for ANG (ab99970; Abcam, San Francisco, CA) according to manufacturer's instructions and read using a microplate reader (Synergy HT, BioTek, Winooski, VT USA). All reactions were performed in triplicate.

### MMP2 zymograph

MMP2 zymograph were performed as previously described [38]. Areas showing enzyme activity show up as regions of negative staining.

### Regional DNA methylation analysis

DNA extraction was performed using Ultra Clean Tissue & Cells DNA Isolation Kit according to manufacturer's instruction (Mo Bio Laboratories, Carlsbad, CA). Methylation status of CpG islands of the MMP2 promoter was assessed by MethylScreen technology using the Epitect methyl II PCR assay (Qiagen) according to manufacturer's instruction. Percentages of unmethylated and hypermethylated CpG values were calculated using a quantitation algorithm provided by the manufacturer (EpiTect Methyl II PCR Assay Handbook - Qiagen).

### Illumina 450 K methylation microarray

DNA extraction was performed from UROtsa^ANG^, UROtsa^Empty^, RT112^KD-ANG^ and RT112^Scr^ cells according to manufacturer's instruction (Ultra Clean Tissue & Cells DNA Isolation Kit, Mo Bio Laboratories). After bisulfite treatment, using EZ DNA methylation kit (Zymoresearch, USA) the whole genome was amplified, enzymatically fragmented and hybridized to the Illumina Infinium Human Methylation 450 Bead Chip kits (Illumina, Inc., San Diego, USA). Following hybridization, allele specific single-base extension and staining were performed and then the BeadChips were imaged on Illumina Bead Array Reader. The image intensities were extracted using Illumina's Bead Scan software (Illumina iScan scanner). The fluorescent signals from methylated and unmethylated alleles represented methylation data point from which the background intensity was then subtracted. Array data export processing and analyses were performed using Illumina GenomeStudio v2011.1 (Methylatioin Module v1.9.0) and the statistical computing package R 3.0.2 (http://www.r-project.org). The statistical significance of the expression data was determined using the fold-change. Hierarchical cluster analysis was performed using complete linkage, and Euclidean distance was used as a measure of similarity. The bisulfite treatment, microarray service and analysis of the array were provided by Macrogen Inc. (Seoul, South Korea). The results from the microarray can be accessed on the Geo-NCBI database, accession number GSE69463.

### RT^2^ profiler PCR Arrays for angiogenesis

RNA was extracted and converted to cDNA as described above. RT^2^ Profiler ‘human angiogenesis’ PCR array (Catalog # PAHS-024ZA; SA Biosciences) was analyzed according to the manufacturer's instruction (www.sabiosciences.com/pcrarraydataanalysis.php) using MyiQ2 Two-Color Real-Time PCR Detection System (Bio-Rad). Relative fold changes in mRNA levels were calculated after normalization to housekeeping control gene targets using the comparative Ct method.

### DNA and RNA from human samples

Total DNA and RNA were extracted and purified from 78 human bladder tumor samples using AllPrep DNA/RNA/Protein Mini Kit (Catalog # 8004, Qiagen) according to manufacturer's instruction.

### Statistical analysis

Disease-free survival (DFS) curves were obtained using the Kaplan-Meier method, and compared by the log-rank test for each prognostic variable. Variables with effect on survival in univariate analysis were included in the Cox proportional hazard regression model. Multivariate analysis was performed to identify independent prognostic variables using a stepwise Cox proportional hazards regression model. Statistical analysis was performed with SPSS. A *p* value less than 0.05 was considered significant.

### Study approval

Institutional Review Board approval at the University of Hawaii Cancer Center was obtained prior to analyses of human bladder tumors.

## SUPPLEMENTARY MATERIAL TABLES AND FIGURE


