# Supplementary material for: Angiogenin contributes to bladder cancer tumorigenesis by DNMT3b-mediated MMP2 activation
Source: Oncotarget. 2016 Jun 15;7(28):43109–23. doi: 10.18632/oncotarget.10097 (PMC5190012; doi:10.18632/oncotarget.10097)
Supplement: Supplementary file 1 [file oncotarget-07-43109-s001.pdf]

Angiogenin contributes to bladder cancer tumorigenesis by DNMT3b-mediated MMP2 activation

Supplementary Material

METHODS

Immunoblotting Antibodies

For imunoblots proteins were transferred to polyvinyl difluoride membranes membranes (Bio-Rad) and stained using a mouse anti-human ANG antibody (C-1, dilution 1:200; Santa Cruz Biotechnology), rabbit anti-human MMP2 antibody (H-76, dilution 1:500, Santa Cruz Biotechnology), rabbit anti-human DNMT1 antibody (#5032, dilution 1:750, Cell Signaling Technology), rabbit anti-human DNMT3a antibody (sc-20703, dilution 1:200, Santa Cruz Biotechnology), goat anti-human DNMT3b antibody (sc-10236, dilution 1:150, Santa Cruz Biotechnology), goat anti-human β-actin antibody (sc-1615, dilution 1:2000,Santa Cruz Biotechnology).

Quantitative reverse transcriptase-PCR

List of Primers:

Gene	Forward Primer	Reverse primer	Size of Product (bp)
**Beta-actine**	ATGATGATATCGCCGCGCTC	CACGATGGAGGGGAAGACG	101
**ANG**	ATTCATGGCAACAAGCGCAG	CTTGCAAGTGGTGACCTGGA	111
**MMP2**	CCTGCAAGTTTCCATTCCGC	CTTCTTGTCGCGGTCGTAGT	116
**DNMT1**	GAGGCAGATGACGATGAGG	CAGTCTTGACGGCTTCTCC	130
**DNMT3a**	AATTCGAGGGCTCCTTGTGG	TTTGCTCCAGGTGGGGTTTT	80
**DNMT3b**	GGGACATCTCACGGTTCCTG	AGCCAAAGATCCTGTTCATCCC	120

Supplemental Table 1: Correlation of methylation status and gene expression. Integration of PCR array and Illumina whole genome methylation array data were integrated in order to correlate the methylation status to gene expression status of 19 targets that were consistently associated with ANG in UROtsa and RT 112 cell lines.

Gene	Methylation Status UROtsa +ANG	Methylation Status RT112 -ANG	Expression Status UROtsa +ANG	Methylation Status RT112 -ANG
**MMP2**	Hypo (Promoter)	Mixed	Overexpression	Underexpression
**MMP14**	Hypo (Promoter)	Mixed	Overexpression	Underexpression
**TGFB1**	Hypo (Promoter)	-	Overexpression	Underexpression
**FN1**	Hypo (Promoter)	Hyper (Promoter)	Overexpression	Underexpression
**NRP1**	Hypo (Promoter)	Hyper (Promoter)	Overexpression	Underexpression
**PGF**	Hypo (Promoter)	-	Overexpression	Underexpression
**MMP9**	Hypo (Promoter)	Hyper (Promoter)	Overexpression	Underexpression
**Heparanase**	Hypo (Promoter)	-	Overexpression	Underexpression
**TGFB2**	Hypo (Promoter)	Hyper (Promoter)	Overexpression	Underexpression
**FGF2**	Hyper (Promoter)	-	Underexpression	Overexpression
**TIMP2**	Hypo (Body)	Hyper (Body)	Underexpression	Overexpression
**VEGFA**	-	-	Underexpression	Overexpression
**TIE1**	Hypo (Body)	Hyper (Body)	Underexpression	Overexpression
**SERPINF1**	Hyper (Promoter)	Hyper (Promoter)	Underexpression	Underexpression
**VEGFB**	Hyper (Promoter)	-	Underexpression	Overexpression
**TIMP3**	Hyper (Promoter) and Hypo (Body)	Hyper (Promoter)	Underexpression	Overexpression
**IFNG**	Hyper (Promoter)	-	Underexpression	Overexpression
**KDR**	Hypo	-	Underexpression	Overexpression

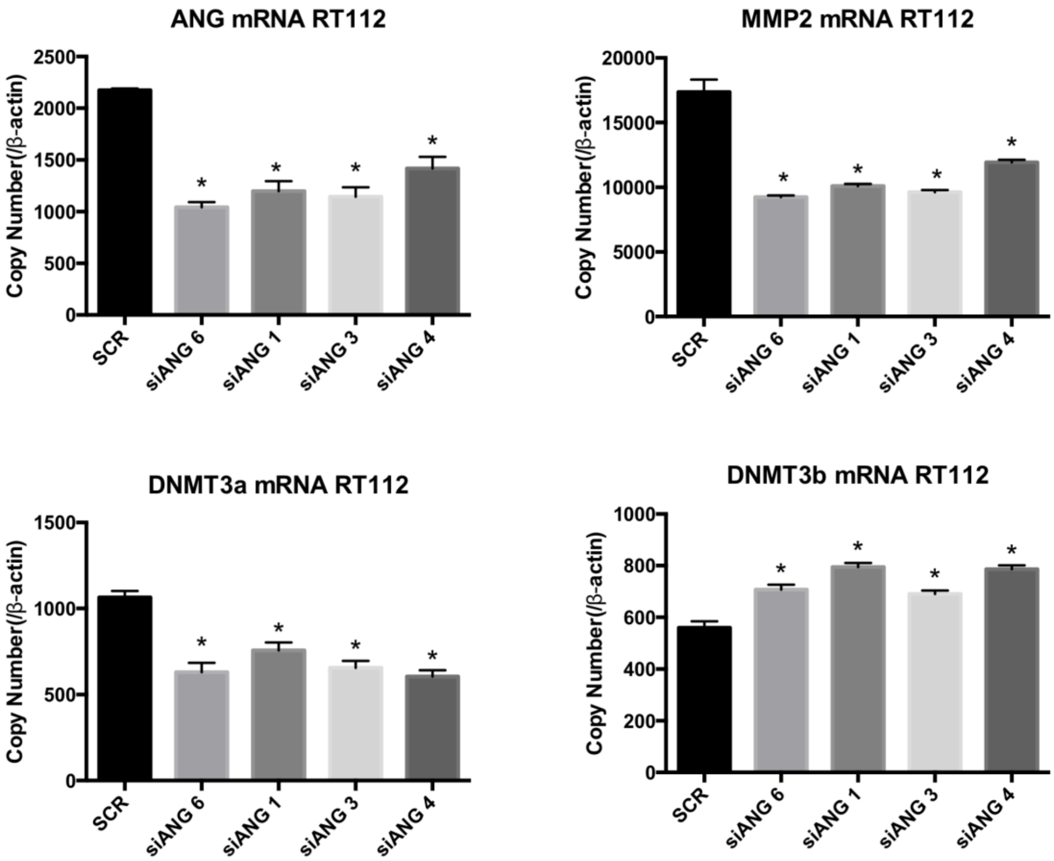

Supplemental Figure 1. RT112 cells were transiently transfected with negative control siRNA ($RT112^{Scr}$) or 4 different siRNA directed at ANG mRNA. The siRNA 6 was the one used for all other experiments.

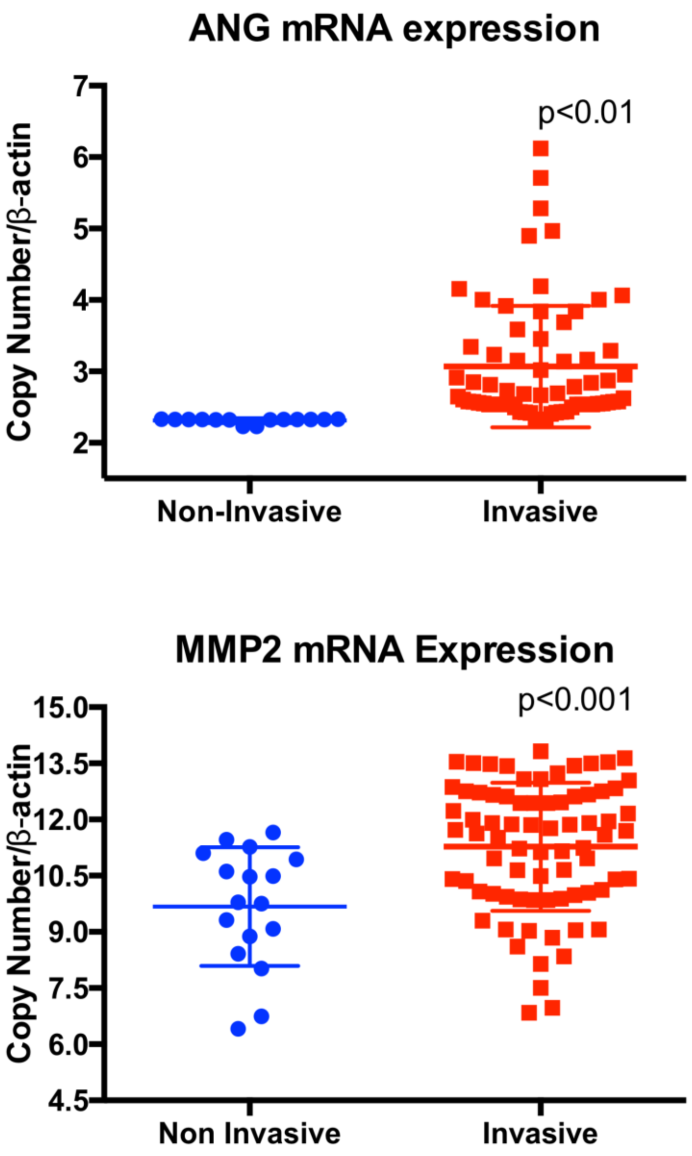

Supplemental Figure 2. mRNA expression of the ANG and MMP2 genes in human bladder cancer from public available dataset. One large public dataset (GDS4456; [1, 2]was obtained from the GEO website (http://www.ncbi.nlm.nih.gov/sites/GDSbrowser?acc=GDS4456) and analyzed for ANG and MMP2 expression. Mean relative mRNA levels for both ANG and MMP2 in muscle invasive bladder cancer were significantly elevated compared to the mean relative mRNA levels for ANG and MMP2 in non-muscle invasive bladder cancer.

References:

1. Riester M, Taylor JM, Feifer A, Koppie T, Rosenberg JE, Downey RJ, Bochner BH and Michor F. Combination of a novel gene expression signature with a clinical nomogram improves the prediction of survival in high-risk bladder cancer. Clin Cancer Res. 2012; 18(5):1323-1333.
2. Riester M, Werner L, Bellmunt J, Selvarajah S, Guancial EA, Weir BA, Stack EC, Park RS, O'Brien R, Schutz FA, Choueiri TK, Signoretti S, Lloreta J, Marchionni L, Gallardo E, Rojo F, et al. Integrative analysis of 1q23.3 copy-number gain in metastatic urothelial carcinoma. Clin Cancer Res. 2014; 20(7):1873-1883.
